# Prenatal Naproxen Reprograms Histopathological and Molecular Facets of the Sex-Based Lung Injury in Adult Offspring of Preeclamptic Rats

**DOI:** 10.3390/ijms27083653

**Published:** 2026-04-20

**Authors:** Sherien A. Abdelhady, Reem H. Elhamammy, Mohamed H. Noureldin, Yasmine Shahine, Nevine M. El-Deeb, Mahmoud M. El-Mas

**Affiliations:** 1 Department of Pharmacology and Biochemistry, Faculty of Pharmacy, Horus University in Egypt, New Damietta 21521, Egypt; shabdelhady@horus.edu.eg; 2 Department of Biochemistry, Faculty of Pharmacy, Alexandria University, Alexandria 21526, Egypt; 3 Department of Biochemistry, Division of Clinical and Biological Sciences, College of Pharmacy, Arab Academy for Science, Technology and Maritime Transport, Alexandria 21625, Egypt; mohamed.noureldin@aast.edu; 4 Department of Microbiology and Immunology, Faculty of Pharmacy, Pharos University in Alexandria, Alexandria 21526, Egypt; yasmine.shahine@pua.edu.eg; 5 Department of Pathology, Faculty of Medicine, Alexandria University, Alexandria 21526, Egypt; nivine.aldeeb@alexmed.edu.eg; 6 Department of Pharmacology and Toxicology, Faculty of Pharmacy, Alexandria University, Alexandria 21526, Egypt; 7 Department of Pharmacology and Toxicology, College of Medicine, Kuwait University, Shadadiya 13060, Kuwait

**Keywords:** preeclampsia, lung offspring, naproxen, Axl, Gas6, apoptosis

## Abstract

Offspring of preeclamptic (PE) mothers are at increased risk of end-organ damage. Given the widespread use of NSAIDs during pregnancy and their reported ability to mitigate organ damage in PE mothers, this study examined whether prenatal naproxen modifies PE-induced lung injury in male and female offspring. PE was induced by orally administered L-nitro-arginine-methyl ester (L-NAME, 50 mg/kg/day for 7 days) to mothers prior to labor, and lung tissues were excised from 3-month-old offspring. Histopathology revealed increased interstitial inflammation and fibrosis in PE versus non-PE offspring lungs. This was more prominent in male than in female PE offspring and was coupled with more pulmonary expression of Axl tyrosine kinase receptor and downstream interleukin-1α (IL-1α) and antiangiogenic Fms-Like Tyrosine Kinase-1(sFlt1) effectors. These sex-related defects disappeared in offspring of PE dams treated prenatally with naproxen (1 mg/kg/day for 7 days). Further, PE offspring exhibited elevations in other inflammatory cytokines, IL-2 and TNFα, and apoptotic markers, caspase-3 and caspase-cleaved cytokeratin 18 (M-30) and total soluble cytokeratin 18 (M-65). The latter effects were evenly seen in both sexes and similarly offset by naproxen. These findings implicate Axl/IL-1α/sFlt1 signaling in the greater lung injury in male PE offspring and suggest a protective effect of gestational naproxen therapy.

## 1. Introduction

Preeclampsia (PE) is an obstetric complication that impacts both the mother and fetus, causing multi-organ damage due to imbalances between proinflammatory and regulatory cytokines [1]. The consequences of PE include disruptions in renal hemodynamics and excretory functions [2], as well as cardiovascular and pulmonary irregularities [3,4], with these effects frequently extending to the offspring [5,6]. Maternal PE was found to increase the risk of respiratory distress syndrome in infants [5,6]. Further, offspring born to mothers with gestational hypertensive disorders exhibited a higher incidence of pneumonia compared with those from normotensive pregnancies [7]. Preeclampsia disrupts multiple essential biological processes, including angiogenesis, inflammation, apoptosis, and autophagy [8,9]. These disturbances are believed to be associated with the dysregulation of several interconnected signaling mechanisms, such as the toll-like receptor 4/nuclear factor kappa-light-chain-enhancer of activated B cells pathway, and the growth-arrest-specific 6/AXL receptor tyrosine kinase (Gas6/AXL) pathway. The disruption of these signaling pathways is associated with a cascade of physiological disturbances that might underlie the widespread maternal and fetal complications characteristic of PE [4,10,11,12,13].

Nonsteroidal anti-inflammatory drugs (NSAIDs) are frequently used during pregnancy as anti-inflammatories, analgesics and antipyretics [14]. Evidence regarding the cardiovascular effects of gestational NSAID use in PE women remains inconsistent. Earlier reports demonstrated that indomethacin administration in women with preeclampsia may trigger significant rises in blood pressure and reduce the effectiveness of antihypertensive therapies, resulting in acute hypertensive crises in some cases and placing maternal health at considerable risk [15,16]. By contrast, more recent randomized controlled trials have re-evaluated the hypertensive effects of NSAIDs and found no significant link between their use and elevated blood pressure in women with PE or other pregnancy-related hypertensive disorders [17]. Moreover, experimental studies have shown that gestational administration of NSAIDs mitigates cardiac, hepatic and renal injuries induced by preeclampsia in rats [18,19,20], suggesting potential beneficial effects of NSAIDs in reducing maternal organ dysfunction associated with the disease. Beyond PE, studies in mice have shown that NSAIDs can suppress bleomycin-induced lung inflammation and fibrosis [21] and spontaneous lung adenocarcinoma formation [22].

Despite the reports that certain maternal health complications associated with PE can improve following antenatal administration of NSAIDs in rats [18,19,20] and that PE-related pathologies can be transmitted to the offspring [5,6], it is still unclear if prenatal NSAID administration can lessen these persistent offspring deficits. In addition, the possibility that such intergenerational effects may differ between male and female offspring has not been explored. To address these gaps, the present study evaluated whether gestational exposure to the NSAID naproxen can mitigate the severity of PE-induced lung injury in adult offspring and whether the naproxen’s action is sexually differentiated. Molecular analyses were also undertaken to delineate the contribution of Gas6/Axl receptor signaling and its downstream inflammatory, antiangiogenic, and apoptotic cascades to the offspring’s pulmonary response to PE and naproxen exposure.

## 2. Results

### 2.1. Effect of Prenatal Naproxen on Lung Histopathology

**Figure 1:**
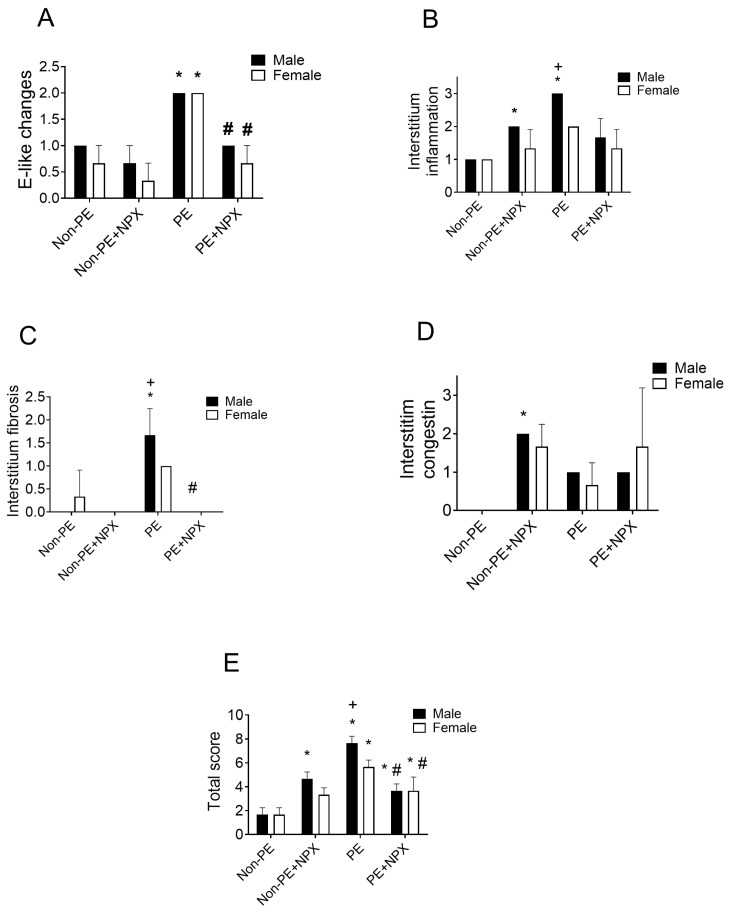
Effects of prenatal naproxen treatment on lung histopathology (E-Like changes, panel (**A**); interstitium inflammation, panel (**B**); interstitium fibrosis, panel (**C**); interstitium congestion, panel (**D**); total histopathology score, panel (**E**)) in 12-week-old male and female offspring of preeclamptic mothers. Data are presented as mean ± SD (n = 3). Comparison between groups was analyzed using Šídák’s multiple comparisons test. Data are compared with non-PE (*) and PE (#) and between males and females (+) at *p* < 0.05. E-Like changes, emphysema-like changes; PE, preeclampsia; NPX, naproxen.

**Figure 2:**
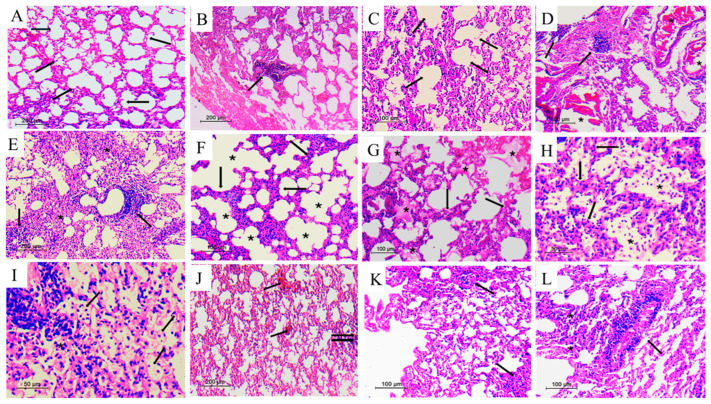
Lung histopathology of male offspring. (**A**) (Non-PE): lung showing intact alveoli, with thin walls and empty alveolar spaces (arrows) (H&E×100). (**B**–**D**) (Non-PE+NPX): (**B**), lung showing intact alveoli, with thin walls and empty alveolar spaces—a focal aggregate of lymphocytes is noted (arrow) (H&E×100); (**C**), lung showing intact alveoli—the alveolar spaces appear empty, and no inflammatory cells, debris or fibrin can be seen (arrows) (H&E×200); (**D**), lung showing congested blood vessels (asterisks)—interstitial lymphoid aggregates are noted (arrows), and the alveoli are intact, with empty alveolar spaces (lower right) (H&E×100). (**E**–**I**) (PE): (**E**), lung showing evident interstitial inflammation (asterisks), with formation of lymphoid aggregates (arrows) (H&E×100); (**F**), lung showing evident interstitial inflammation—the alveolar septa are thick, densely infiltrated by inflammatory cells (arrows), and emphysema-like changes are evident, with fusion of alveolar spaces (asterisks) (H&E×200); (**G**), fibrin is noted within the alveolar spaces (asterisks)—the alveolar septa are thick and infiltrated by inflammatory cells (arrows) (H&E×200); (**H**), inflammatory cells and cell debris are seen within the alveolar spaces (asterisks). The alveolar septa are thick and infiltrated by inflammatory cells (arrows) (H&E×400); (**I**), inflammatory cells (arrows) are seen within the alveolar spaces. An interstitial lymphoid aggregate is noted (upper left) (H&E×400). (**J**–**L**) (PE+NPX): (**J**), the alveoli are largely intact—congested blood vessels are seen (arrows), as well as a focus of interstitial inflammation (double arrows) (H&E×100); (**K**), the alveoli appear intact, and some foci show thickened alveolar septae, infiltrated by inflammatory cells (arrows) (H&E×200); (**L**), the alveoli are largely intact—apart from few alveoli showing thickening of the alveolar septae (asterisks), an interstitial lymphoid aggregate is seen (arrow) (H&E×200).

Histological examination of H&E-stained lung sections revealed that PE induced marked pathological alterations, which included interstitial inflammation (Figure 1B) and fibrosis (Figure 1B), with additional evidence of interstitial congestion in the male PE group (Figure 1D). The overall histopathological score, which reflects cumulative tissue damage, was significantly elevated in both male and female offspring of the PE group compared with the non-PE controls, with male offspring exhibiting more severe changes than females (Figure 1E). The prenatal administration of naproxen significantly reduced the PE-induced lung injuries and eliminated the sex-related differences observed in the total histopathology score (Figure 1E). Male, but not female, offspring of naproxen-treated non-PE rats exhibited significant increases in interstitial inflammation and congestion (Figure 1B,D), accompanied by a significant rise in the total histopathology score (Figure 1E). Representative photomicrographs of these morphological findings are presented in Figure 2. Raw histopathology data of this experiment are provided in Supplementary Files S1 and S2.

### 2.2. Effect of Prenatal Naproxen on Inflammatory and Antiangiogenic Factors

**Figure 3:**
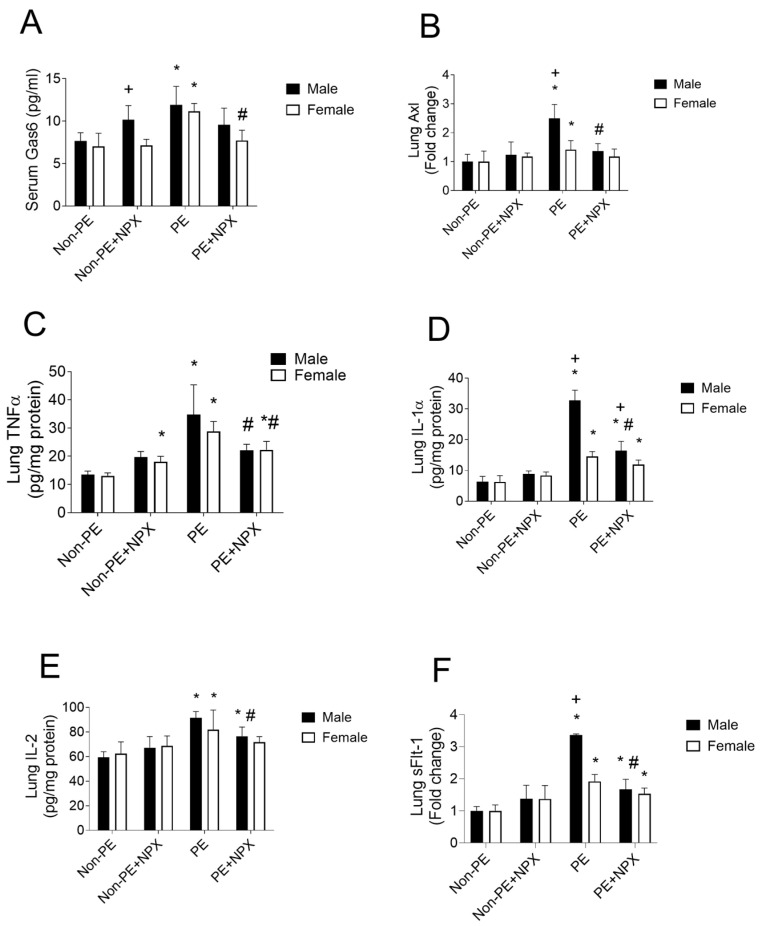
Effect of prenatal naproxen treatment on serum Gas6 (panel (**A**)), lung Axl (panel (**B**)), lung TNFα (panel (**C**)), lung IL-1α (panel (**D**)) and lung IL-2 (panel (**E**)) and lung sFlt (panel (**F**)) in male and female offspring of control (non-PE) and PE rats. Values are expressed as mean ± SD (n = 5). Lung Axl and sFlt-1 levels are expressed as fold changes relative to the control (non-PE) group. Lung Axl and sFlt-1 levels are expressed as fold changes relative to the control (non-PE) group. Comparisons between groups were analyzed using 3-way ANOVA (GraphPad Prism v7.0), followed by Šídák’s multiple comparisons test. Data are compared with non-PE (*), PE (#), and between males and females (+) at *p* < 0.05. Non-PE, non-preeclamptic; PE, preeclamptic; NPX, naproxen, Gas6, growth arrest-specific 6; Axl, Axl receptor tyrosine kinase; TNF-α, tumor necrosis factor alpha; IL-1α, interleukin 1 alpha; IL-2, interleukin 2; sFlt, soluble fms-like tyrosine kinase-1.

Figure 3 illustrates the effects of naproxen on Gas6/Axl signaling and associated inflammatory and antiangiogenic indicators. No sex-related differences were observed in these biomarkers among adult offspring of non-PE rats. Both male and female PE offspring showed significant elevations in serum Gas6 levels (Figure 3A) and lung concentrations of inflammatory mediators, including Axl (Figure 3B), TNF-α (Figure 3C), IL-1α (Figure 3D), and IL-2 (Figure 3E), compared with their non-PE counterparts. In addition, the gene expression of the antiangiogenic factor sFlt-1 in lung tissue was significantly upregulated in PE offspring of both sexes (Figure 3F). The increases in Axl, IL-1α, and sFlt-1 were more pronounced in male than in female progeny.

Prenatal naproxen treatment significantly attenuated the PE-induced molecular changes, reducing inflammatory marker levels toward control values (Figure 3). Interestingly, while naproxen significantly reduced sFlt-1 expression in male lungs, this effect was not observed in females (Figure 3F). Conversely, naproxen administration during normal pregnancies did not significantly alter the studied markers in either sex; however, significantly higher levels of Gas6 were demonstrated in male offspring compared with females (Figure 3A). Raw data of this experiment are provided in Supplementary File S3.

### 2.3. Effect of Naproxen on Apoptotic Markers

**Figure 4:**
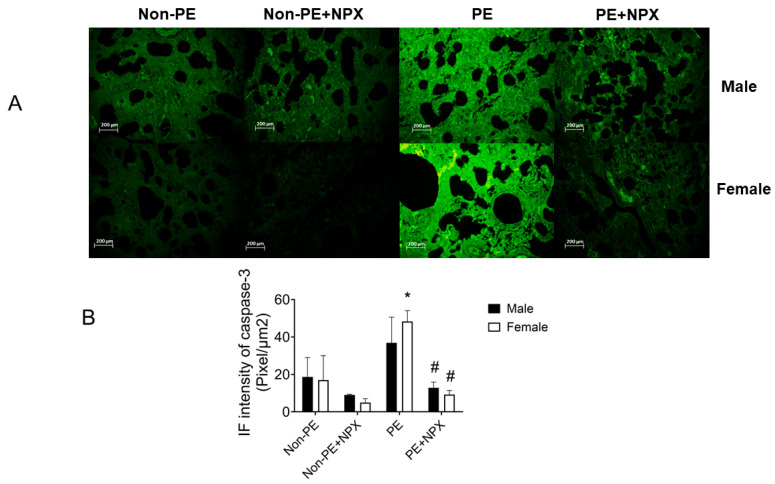
Effect of prenatal naproxen treatment on pulmonary caspase-3 immunofluorescence in male and female offspring of control (non-PE) and PE rats. Values are expressed as mean ± SD (n = 5, (**B**)). Representative photomicrographs are shown in (**A**). Comparisons between groups were analyzed using 3-way ANOVA, Šídák’s multiple comparisons test. Data are compared with non-PE (*), PE (#) at *p* < 0.05. Non-PE, non-preeclamptic; PE, preeclamptic; NPX, naproxen.

As depicted in Figure 4, immunofluorescence analysis of the apoptotic marker caspase-3 showed no significant difference in apoptosis levels between male and female rats in the non-PE group (*p* > 0.9999). In contrast, caspase-3 expression was significantly elevated in PE-derived female offspring (*p* = 0.0067), whereas the increase observed in PE-derived male offspring did not reach statistical significance (*p* = 0.1724). Prenatal naproxen treatment markedly reduced caspase-3 expression in both male and female offspring compared with the PE group (*p* = 0.0114 and *p* = 0.0033, respectively). Furthermore, no significant sex-related differences were detected in caspase-3 expression within either of the prenatal naproxen treatment groups (*p* = 0.9990 for both comparisons).

**Figure 5:**
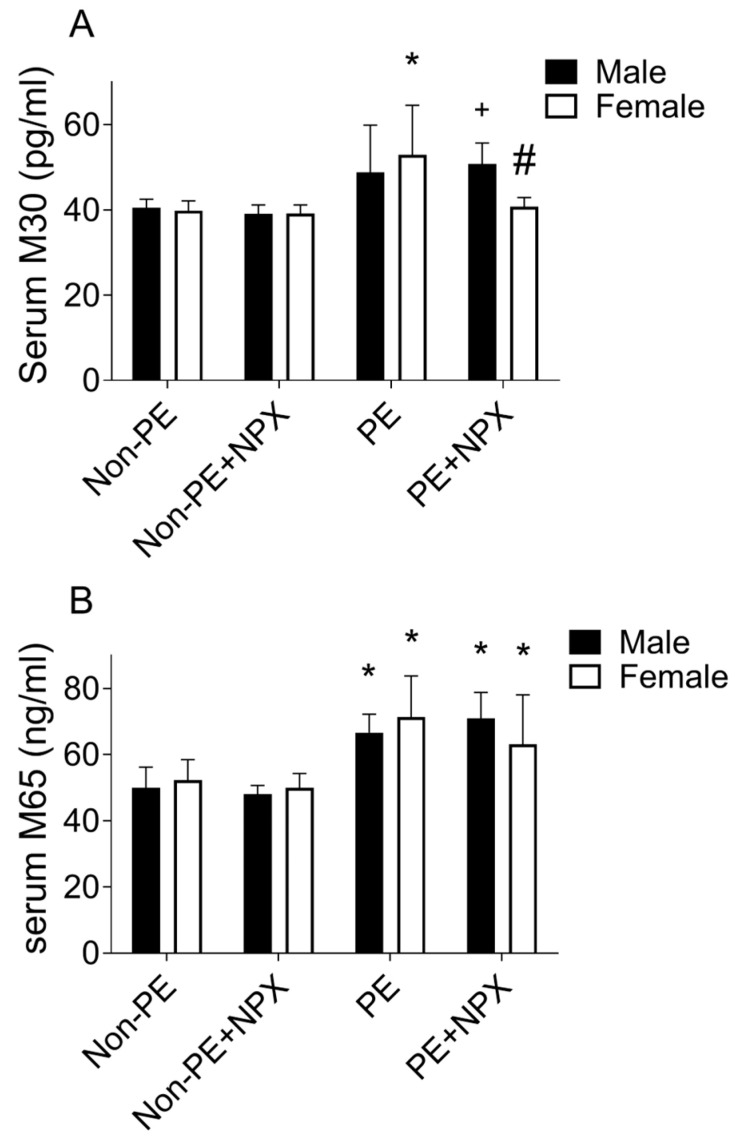
Effect of prenatal naproxen on serum levels of M-30 (panel (**A**)) and M-65 (panel (**B**)) in male and female offspring of control (non-PE) and PE rats. Values are expressed as mean ± SD (n = 5). Comparisons between groups were analyzed using 3-way ANOVA, followed by Šídák’s multiple comparisons test. Data are compared with non-PE (*), PE (#) and between males and females (+) at *p* < 0.05. Non-PE, non-preeclamptic; PE, preeclamptic; NPX, naproxen. M-30, cytokeratin 18-M-30; M-65, cytokeratin 18-M65.

Serum levels of CK18-based biomarkers M-30 and M-65 were measured to evaluate cell death. PE induced a significant increase in serum M-65 levels (Figure 5B) in both male and female offspring compared with their respective non-PE controls. In contrast, elevated M-30 levels (Figure 5A) were observed only in female offspring of PE mothers. Prenatal naproxen treatment did not significantly alter M-30 or M-65 levels in non-PE offspring of either sex compared with controls. Among PE-derived offspring prenatally treated with naproxen, M-65 levels remained comparable to naproxen-untreated PE counterparts in both sexes (Figure 5B). M-30 levels were markedly reduced only in female offspring, showing significantly lower values than both PE-derived females and prenatally naproxen-treated PE-derived males (Figure 5A). Raw data of this experiment are provided in Supplementary File S3.

## 3. Discussion

This study evaluated the impact of PE on lung structure and key molecular signaling pathways in adult rat offspring of both sexes and determined whether prenatal administration of the nonsteroidal anti-inflammatory drug naproxen could modulate these effects. Offspring from PE dams exhibited pronounced pulmonary histopathological abnormalities, including interstitial inflammation, fibrosis, and vascular congestion, along with significant upregulation of Gas6/Axl signaling and its downstream proinflammatory, antiangiogenic, and apoptotic mediators. Although these pathological and molecular alterations were observed in both offspring sexes, they were more prominent in males, indicating a clear sexual dimorphism in PE-associated pulmonary injury. Antenatal naproxen treatment markedly alleviated PE-induced lung injury, improved the associated molecular abnormalities, and abolished the sex-related differences in PE outcomes. Collectively, these observations suggest that prenatal naproxen may offer a therapeutic strategy against long-term PE-induced pulmonary damage and its sex-specific consequences in the offspring.

Data from the present study demonstrated that male offspring of PE dams were more vulnerable to lung injury, exhibiting greater inflammation and fibrosis than their female counterparts. Nevertheless, the overall histological scores confirmed that females also display significant architectural lung changes. These observations are consistent with previous studies reporting impaired lung growth in male rodents, as indicated by higher mean linear intercept and lower radial alveolar counts in hyperoxia-induced lung injury models [23]. Similarly, maternal high-salt diet exposure has been shown to trigger pulmonary fibrosis in both sexes, with males exhibiting more severe fibrosis [24]. In this regard, gonadal sex hormones may contribute to the observed sex-specific effects in PE offspring. For example, estrogen is believed to promote alveolar development, regeneration, and protection during the active growth phase [25] and contribute to the earlier production of surfactant in female fetal lungs [26]. Conversely, androgens function to delay lung maturation by downregulating glucocorticoid receptor mRNA and protein expression [27] and promoting proliferation of epithelial, endothelial, and mesenchymal cells in the developing lung [28].

Molecular studies were undertaken to determine the roles of the Gas6/Axl pathway and downstream inflammatory signals in the sex-related pulmonary defects in PE offspring. Recent evidence points to a causal role of Gas6/Axl signaling in the development of both placental and systemic end-organ pathologies associated with PE. This concept is supported by the findings that (i) parenteral administration of Gas6 to pregnant rats enhances Axl signaling and recapitulates key clinical and pathological features of PE, including trophoblast apoptosis, hypertension, and proteinuria, and (ii) pharmacological inhibition of Axl using R428 markedly attenuates most of these PE-associated abnormalities [4,29,30,31]. Although its pathogenic role in maternal PE is well established [29,32], direct evidence linking Gas6/Axl signaling to PE-related complications in the offspring remains scarce. Our study addresses this gap by examining whether Gas6/Axl signaling contributes to the lung injury observed in adult rat offspring of PE dams. We observed significant elevations in Gas6/Axl signaling and associated inflammatory mediators (TNFα, IL-1α, IL-2), with markedly higher increases in Axl and IL-1α in males compared with females. The observed upregulation of the inflammatory Axl/IL-1α axis alongside the more severe pulmonary structural injury in male offspring suggests a possible link, though a causal relationship cannot be ascertained.

Another principal aim of the current study was to determine whether prenatal exposure to the nonsteroidal anti-inflammatory drug naproxen could modulate PE-induced pulmonary injury in adult rat offspring and contribute to the sexual dimorphism in this interaction. We have recently reported that antenatal naproxen administration to pregnant rats ameliorates renal and hepatic structural abnormalities in weaning PE dams while simultaneously suppressing the hyperactivated inflammatory, oxidative, fibrotic, and autophagic pathways [18,19]. Although fetal programming associated with PE is well recognized as a critical determinant of increased vulnerability to end-organ injury in the developing offspring [33,34,35], evidence addressing whether prenatal naproxen affects offspring organ damage has been lacking. The present study is the first to reveal that prenatal naproxen markedly alleviated interstitial inflammation and fibrosis in the lungs of PE offspring and blunted the accompanying exaggeration in the inflammatory Axl/IL-1α signaling. Further, naproxen reduced both histological and molecular indicators of lung injury to comparable levels in male and female offspring, suggesting a key role for the Axl/IL-1α cascade in the aggravated vulnerability of the male offspring to the lung injurious effect of PE.

Interestingly, in contrast to its apparent protective effects in offspring of PE dams, prenatal naproxen exposure in non-PE pregnancies was associated with increased interstitial inflammation and vascular congestion, along with a higher total histopathology score, specifically in male but not female offspring (see Figure 1). This sex-dependent structural alteration suggests a differential sex susceptibility to naproxen under non-pathological conditions. Notably, these changes were not accompanied by detectable alterations in Axl/IL-1α signaling compared with control non-PE rats, indicating that the observed tissue effects may be mediated through alternative pathways. These findings highlight a potentially complex and context-dependent action of naproxen and underscore the need for further investigations to elucidate the molecular mechanisms underlying the male-specific pulmonary structural changes caused by naproxen in offspring of non-PE dams.

Given the key contribution of Gas6/Axl signaling to angiogenic imbalance in PE [36], we explored the presumption that the antiangiogenic factor sFlt-1 contributes to the naproxen modulation of PE-induced lung injury in the offspring. This investigation was prompted by evidence that (i) inflammatory cytokines stimulate the expression of antiangiogenic factors such as sFlt-1 in trophoblasts and placental tissues [11] and (ii) elevated sFlt-1 levels in preeclamptic amniotic fluid disrupt VEGF-dependent angiogenesis, thereby impairing fetal lung development and maturation [37,38,39]. Consistent with these findings, our data revealed a marked increase in pulmonary sFlt-1 gene expression in lungs of both male and female PE offspring, with males exhibiting significantly higher levels. More importantly, prenatal naproxen therapy effectively counteracted this elevation and restored sFlt-1 expression to comparable levels in the two sexes. These observations suggest that naproxen favorably impacts the pulmonary phenotype of PE offspring via the suppression of the overactivation of the Axl/IL-1α/sFlt-1 axis, thereby mitigating the antiangiogenic and inflammatory disturbances associated with PE-induced fetal programming.

Cell death dysregulation is increasingly recognized as a key mechanism in preeclamptic fetal programming, predisposing offspring to long-term structural and functional organ impairments [40]. In particular, excessive apoptosis and necrosis during fetal development can disrupt alveolarization and vascular formation, thereby predisposing to chronic pulmonary injury [41]. In this study, the similar elevations in caspase-3 immunofluorescence and serum M-30 and M-65 levels in both male and female offspring of preeclamptic (PE) rats suggest that activation of apoptotic and necrotic pathways may contribute to the programming of PE-induced lung injury in both sexes. Notably, caspase-3 and M-30 are widely recognized to mark apoptotic activity, whereas M-65 denotes both apoptosis and necrosis [42,43]. Additionally, the ability of prenatal naproxen to reduce the elevated caspase-3 and M-30 without significantly altering M-65, infers selective suppression of apoptosis, while non-apoptotic cell death, likely necrosis, may still persist. This antiapoptotic action, together with naproxen’s ability to counteract inflammatory and antiangiogenic biomarkers, highlights the molecular basis of its protective effect against PE-induced pulmonary injury.

Notably, naproxen was selected in the present study based on evidence of its superior efficacy, compared with other NSAIDs, e.g., celecoxib and diclofenac, in ameliorating end-organ damage and underlying molecular alterations in PE rats [18,19,20]. In this context, two points regarding the naproxen choice warrant clarification. First, the use of NSAIDs during pregnancy should be discussed in relation to current recommendations for aspirin in PE prevention. Low-dose aspirin is widely recommended for women at high risk of PE due to its antiplatelet and vasculoprotective effects, primarily mediated through the selective inhibition of thromboxane A_2_ synthesis and improvement of uteroplacental perfusion [44,45]. This contrast with conventional NSAIDs such as naproxen that elicit broader anti-inflammatory effects via COX inhibition. Further studies are required to evaluate the effectiveness of gestational aspirin in reducing organ damage in offspring of PE pregnancies.

Second, although naproxen and other NSAIDs are commonly used during pregnancy and are classified as relatively safe for mothers and fetuses, epidemiological studies suggest that they may increase the risk of spontaneous abortion, intrauterine growth retardation, and preterm constriction of ductus arteriosus [46,47]. These adverse effects highlight the importance of exercising caution when interpreting their translational relevance. It should be noted that beyond the demonstration of the pulmonary protective effects of naproxen in this study, a major finding is the upregulation of the Axl/IL-1α/sFlt-1 axis that accompanied the developmental pulmonary toxicity in PE offspring. In light of the established causal relationship between Gas6/Axl signaling and PE-related pathologies [29,30], more investigation is warranted to determine whether targeted downregulation of this pathway would confer therapeutic benefit in mitigating pulmonary toxicity in PE offspring.

While our data demonstrate a suppressive effect of naproxen on the Axl/IL-1α/sFlt-1 axis, it remains unclear whether this effect is related to naproxen inhibition of COX-2–mediated inflammation. Evidence from alveolar epithelial cells indicates that Gas6 stimulation upregulates COX-2 expression and increases COX-2-derived prostaglandins, whereas Axl knockdown attenuates these responses [48]. Additionally, the proinflammatory transcription factor NF-κB, an established driver of COX-2 expression, also functions downstream of Axl signaling in immune and cancer cells [49]. Other studies, however, suggest that Gas6/Axl signaling can modulate inflammation through mechanisms that are not limited to amplification of COX-2–mediated responses [50]. Together, these observations indicate that the interaction between Gas6/Axl and COX-2 is context-dependent, varying according to cell type, stimulus, and the surrounding inflammatory environment.

This study provides evidence that prenatal naproxen therapy attenuates preeclampsia-induced lung injury in offspring and simultaneously suppresses the Axl/IL-1α/sFlt-1 signaling pathway and improves pulmonary angiogenic balance. These changes are accompanied by reduced structural lung damage and diminished male-predominant vulnerability to PE-related pulmonary dysfunction. Clinically, these results highlight the potential of targeting the Axl/IL-1α/sFlt-1 axis to prevent fetal programming of long-term respiratory complications in pregnancies complicated by preeclampsia, offering a promising therapeutic avenue that warrants further translational and clinical investigation.

## 4. Materials and Methods

### 4.1. Animals

Adult female Sprague Dawley rats weighing 170–220 g were used in this research. The rats were acquired from the animal house of the Faculty of Pharmacy, Pharos University in Alexandria, Alexandria, Egypt. Prior to the experiment, rats were housed under observation for a week with free access to water and standard rat pellets ad libitum. All animal procedures in the present study were conducted according to the guidelines of Egypt’s guide for the care and use of laboratory animals [51], Unit of Research Ethics Approval Committee, Pharos University in Alexandria, Egypt (Approval No.: 01202403313208), and with ARRIVE guidelines and the National Institutes of Health, Bethesda, MD, USA, for the care and use of laboratory animals.

### 4.2. PE Induction and Experimental Protocol

Breeding was permitted between adult nulliparous female and male rats (ratio 1:1). The day of conception is recognized by the existence of spermatozoa in the vaginal lavage or a vaginal plug. Starting on day 14 of conception, a daily dose of Nω-nitro-L-arginine methyl ester (L-NAME, 50 mg/kg) was administered via oral gavage for 7 consecutive days for the induction of PE [33,34]. The preeclamptic mother rats were distributed randomly into 4 groups, with 5 rats each: (i) non-preeclamptic pregnant (non-PE)/saline (ii) PE/saline, (iii) PE/naproxen (1 mg/kg/day, Sigma, St. Louis, MO, USA), and (iv) non-PE/naproxen (1 mg/kg/day). Like L-NAME, naproxen was administered via oral gavage for 7 consecutive days, starting from day 14 till labor. The naproxen dose was selected based on prior studies demonstrating its efficacy in mitigating inflammation and organ damage [18,19,20,52]. The offspring were weaned at three weeks postpartum, which is the standard developmental time point when rat pups are sufficiently mature to eat solid food independently [53]. To avoid litter effects, only one offspring per sex per litter was included in the study. Adult 3-month-old offspring were weighed, then grouped into male (4 groups, 5 rats each) and female (4 groups, 5 rats each). In either offspring sex, the body weight was not significantly affected by PE or prenatal naproxen administration (data are shown in Supplementary File S4). Rats were euthanized by i.p. injection of an overdose of thiopental sodium (100 mg/kg, Biochemie GmbH, Vienna, Austria). Blood was collected from the rats’ abdominal aorta and centrifuged (1200× *g* for 10 min at room temperature or 4 °C), and the serum was aspirated and stored at −80 °C for subsequent analyses. Lung tissues were excised and divided into two parts for histopathological and molecular investigations. The first part was immediately frozen in liquid nitrogen and stored at −80 °C, whereas the second part was fixed in 10% formaldehyde. The animal groupings and treatments were randomly assigned to the respective rat groups using the simple randomization sequence, which is based on a single sequence of random assignments. Further, blinding and randomization were applied during histopathology and molecular studies to minimize bias.

### 4.3. Enzyme-Linked ImmunosorbentAssay—(ELISA) Measurements

Serum caspase-cleaved cytokeratin 18; M-30 [Rat CK 18-M30] and serum total soluble cytokeratin 18, M-65 [Rat CK 18-M65], MyBioSource.com. San Diego, CA, USA; serum Gas6 and lung tissues of tumor necrosis factor alpha (TNFα), interleukin-1 alpha (IL-1α), and Interleukin-2 (IL-2), Chongqing Biospes Co., Ltd., Chongqing, China, were determined by ELISA kits according to the manufacturer’s instructions.

### 4.4. Real-Time Reverse Transcriptase–Polymerase Chain Reaction (qRT-PCR)

Total RNA was isolated from lung tissues using RNeasy Mini Kit (Qiagen GmbH, Qiagen, Germany), following the manufacturer’s instructions. The RNA concentration of the samples was determined using nanodrop. cDNA was synthesized from the purified RNA using the QuantiTect Reverse Transcription Kit (Qiagen, Germany). The reaction mixture included RNA and master mix, which were placed at 42 °C and then inactivated at 95 °C. The cDNA was used to quantify the tissues’ gene expression of Soluble Fms-Like Tyrosine Kinase-1 (sFlt-1) and Axl genes by Rotor-Gene Q qRT-PCR using QuantiTect SYBR Green PCR Master Mix. Quantitative PCR amplification conditions started with an initial denaturation for 10 min at 95 °C and then amplification by 40 cycles of PCR as follows: denaturation at 95 °C for 5 s, annealing at 55 °C for 15 s and extension at 60 °C for 15 s. The housekeeping gene 18s rRNA was used as a reference gene for normalization. Primers used for the target gene and the housekeeping gene are presented in Table 1. The values of threshold cycle were determined by Rotor-Gene Q-Pure Detection version 2.1.0 (build 9). For each gene, the relative change in mRNA in samples was normalized to 18 s rRNA using the 2^−ΔΔCt^ method [18,54].

### 4.5. Histopathological Examination

Male and female offspring lung aliquots were dissected, preserved in 10% neutral-buffered formalin, and later embedded in paraffin. The blocks were stained with hematoxylin and eosin (H&E) after being sectioned at 5 µm intervals using a Leica 819 comp microtome (Nussloch, Germany). Morphologic alterations were determined by light microscopy. The following 4 histopathologic features were assessed: (i) emphysema-like changes (airspace enlargement relative to background parenchyma with fragmented alveolar walls), (ii) interstitium inflammation, (iii) interstitium fibrosis, and (iv) interstitium congestion. Each histological feature was scored as follows: 0 denoted the absence of the feature, 1 denoted less than 10%, 2 denoted 10–24%, 3 denoted 25–50%, and 4 denoted more than 50% of the surface area of the sections under examination. The total score was calculated as the sum of values of all the assessed features for each animal. All groups, including the non-PE, were evaluated independently using the same scoring criteria. The scoring was done in a blinded manner in 20 microscopic fields per section (n = 3 animals).

### 4.6. Immunofluorescence Staining for Caspase-3

For caspase-3 immunofluorescence, paraffin sections were prepared and deparaffinized in xylene overnight. The sections were rehydrated in descending alcohol grades and then treated with 1% fetal calf serum in phosphate-buffered saline for antigen retrieval. Sections were coated with caspase-3 monoclonal primary antibody (Thermofisher, Waltham, MA, USA) and incubated overnight at 4 °C in a humid chamber. The secondary antibody AlexaFluor-488 goat anti-mouse IgG (Thermofisher USA) was added, and the mixture was incubated at room temperature for 1 h in the dark. Sections were examined using a Leica fluorescence microscope (Leica Microsystems, Wetzlar, Germany), and caspase-3 intensity was quantified with Image J software version 1.51n (National Institutes of Health, Bethesda, MD, USA) [55,56].

### 4.7. Statistical Analysis

Values were expressed as means ± SD. A two-tailed three-way ANOVA was used to evaluate the effects of multiple factors and their interactions on the results, followed by Šídák’s multiple comparisons test. The GraphPad Prism v7.0 (GraphPad Prism Inc., La Jolla, CA, USA) was utilized for these analyses and statistical significance was defined as *p* < 0.05.

## Figures and Tables

**Table 1:** Primers sequences of studied genes.

Gene Name	Access No.	Primer	Sequence
*sFlt-1*	NM_001309381	Forward	TACGTCACAGATGTGCCAAAC
		Reverse	GCAGTGCTCACCTCTAACGA
*Axl*	NM_031794.2	Forward	CTACGAGACGTCATGGTAG
		Reverse	GCTCTGATCTTGTGCAGATG
*18 s rRNA*	NR_046237.2	Forward	GTAACCCGTTGAACCCCATT
		Reverse	CAAGCTTATGACCCGCACTT

## Data Availability

The original contributions presented in this study are included in the article/Supplementary Materials. Further inquiries can be directed to the corresponding author.

## Supplementary Materials

The following supporting information can be downloaded at: https://www.mdpi.com/article/10.3390/ijms27083653/s1.

## Abbreviations

The following abbreviations are used in this manuscript:

PE	Preeclampsia
L-NAME	L-nitro-arginine-methyl ester
AXL	Axl tyrosine kinase receptor
sFlt-1	Soluble Fms-Like Tyrosine Kinase-1
M-30	Caspase-cleaved cytokeratin 18
M-65	Total soluble cytokeratin 18
JAK	Janus kinase
STAT	Signal transducer and activator of transcription
Gas6	Growth arrest–specific 6
VEGFR-2	Vascular endothelial growth factor receptor 2
PKC	protein kinase C
MEK	mitogen-activated protein kinase/extracellular signal–regulated kinase
NSAIDs	Nonsteroidal anti-inflammatory drugs
TNFα	Tumor necrosis factor alpha
qRT-PCR	Real-time reverse transcriptase-polymerase chain reaction
cDNA	Complementary DNA
IL-1α	Interleukin-1 alpha
IL-2	Interleukin-2
H&E	Hematoxylin and eosin
ELISA	Enzyme-Linked Immunosorbent Assay.
