# Supplementary material for: Prenatal Naproxen Reprograms Histopathological and Molecular Facets of the Sex-Based Lung Injury in Adult Offspring of Preeclamptic Rats
Source: Int J Mol Sci. 2026 Apr 20;27(8):3653. doi: 10.3390/ijms27083653 (PMC13116112; doi:10.3390/ijms27083653)
Supplement: Supplementary file 1 [file ijms-27-03653-s001.zip › Supplementary file S1 (Histology data, Non PE and PE).pdf]

**Non PE:
Male: : [mean alv count. 12]
*206.M:
*Alveolar count: 11
*Alveoli.
Cell debris: . 1
Infl cells: 0
Fibrin:. 0
Thick septa: 1
E- like changes: 1
* Interstitium:
Infl. 1
Fibrosis . 0
Congestion 0
**TOTAL: . 4/24

*209 M:
*Alveolar count: 10
*Alveoli.
Cell debris: . 1
Infl cells: 1
Fibrin:. 0
Thick septa: 1
E- like changes: 1
* Interstitium:
Infl. 1
Fibrosis . 0
Congestion 0
**TOTAL: . 5/24

*243 M:
*Alveolar count: 15
*Alveoli.
Cell debris: . 1
Infl cells: 0
Fibrin:. 0
Thick septa: 1
E- like changes: 1
* Interstitium:
infl. 1
Fibrosis . 0
Congestion 0
**TOTAL: . 4/24

Female: [mean alv count. 13.33]
*206 F:
*Alveolar count: 13
*Alveoli.

Cell debris: . 0
Infl cells: 1
Fibrin:. 0
Thick septa: 1
E- like changes: 0
* Interstitium:
Infl. 1
Fibrosis . 0
Congestion. 0
**TOTAL: . 3/24

*242 F:
*Alveolar count: 11
*Alveoli.
Cell debris: . 1
Infl cells: 1
Fibrin:. 0
Thick septa: 0
E- like changes: 1
* Interstitium:
Infl. 1
Fibrosis . 0
Congestion 0
**TOTAL: . 4/24

*243 F:
*Alveolar count: 16
*Alveoli.
Cell debris: . 1
Infl cells: 0
Fibrin:. 0
Thick septa: 1
E- like changes: 0
* Interstitium:
Infl. 1
Fibrosis . 1
Congestion 0
**TOTAL: . 4/24
—---
** PE
Male: : [mean alv count. 9]
*251 M:
*Alveolar count: 10
*Alveoli.
Cell debris: . 2
Infl cells: 1
Fibrin:. 0
Thick septa: 2

E- like changes: 2
* Interstitium:
Infl.   3
Fibrosis .   2
Congestion.   1
**TOTAL: . 13/24

*252 M:
*Alveolar count: 8
*Alveoli.
Cell debris: .   1
Infl cells: 2
Fibrin:.   2
Thick septa: 2
E- like changes: 2
* Interstitium:
Infl.   3
Fibrosis .   1
Congestion.   1
**TOTAL: . 14/24

Female: [mean alv count. .9.3]
*248 F:
*Alveolar count: 8
*Alveoli.
Cell debris: .   1
Infl cells: 1
Fibrin:.   2
Thick septa: 2
E- like changes: 2
* Interstitium:
Infl.   2
Fibrosis .   1
Congestion.   0
**TOTAL: . 11/24

*252 F:
*Alveolar count: 8
*Alveoli.
Cell debris: .   1
Infl cells: 1
Fibrin:.   1
Thick septa: 1
E- like changes: 2
* Interstitium:
Infl.   2
Fibrosis .   1

Congestion.   1
**TOTAL: . 10/24

*253 F:
*Alveolar count: 12
*Alveoli.
Cell debris: .   1
Infl cells: 2
Fibrin:.   1
Thick septa: 2
E- like changes: 2
* Interstitium:
Infl.   2
Fibrosis .   1
Congestion.   1
**TOTAL: . 12/24.
—---
