# Supplementary material for: Prenatal Naproxen Reprograms Histopathological and Molecular Facets of the Sex-Based Lung Injury in Adult Offspring of Preeclamptic Rats
Source: Int J Mol Sci. 2026 Apr 20;27(8):3653. doi: 10.3390/ijms27083653 (PMC13116112; doi:10.3390/ijms27083653)
Supplement: Supplementary file 1 [file ijms-27-03653-s001.zip › Supplementary file S2 (Histology data, NPX-PE and NPX).pdf]

** PE+NAP:
Male:[mean alv count: 11.66]
*267.M:
*Alveolar count: 11
*Alveoli.
Cell debris: . 0
Infl cells: 1
Fibrin:. 1
Thick septa: 0
E- like changes: 1
* Interstitium:
Infl. 2
Fibrosis . 0
Congestion 1
**TOTAL: . 6/24

*269 M:
*Alveolar count: 10
*Alveoli.
Cell debris: . 0
Infl cells: 1
Fibrin:. 2
Thick septa: 0
E- like changes: 1
* Interstitium:
Infl. 2
Fibrosis . 0
Congestion 1
**TOTAL: . 7/24

*262 M:
*Alveolar count: 14
*Alveoli.
Cell debris: . 0
Infl cells: 0
Fibrin:. 0
Thick septa: 1
E- like changes: 1
* Interstitium:
infl. 1
Fibrosis . 0
Congestion 1
**TOTAL: . 4/24

Female:[mean alv count: 12.33]
*265 F:
*Alveolar count: 16
*Alveoli.

Cell debris: .   0
Infl cells: 1
Fibrin:.   0
Thick septa: 0
E- like changes: 1
* Interstitium:
Infl.   2
Fibrosis .   0
Congestion.   0
**TOTAL: . 4/24

*270 F:
*Alveolar count: 11
*Alveoli.
Cell debris: .   0
Infl cells: 1
Fibrin:.   0
Thick septa: 0
E- like changes: 1
* Interstitium:
Infl.   1
Fibrosis .   0
Congestion   3
**TOTAL: . 6/24

*271 F:
*Alveolar count: 10
*Alveoli.
Cell debris: .   1
Infl cells: 0
Fibrin:.   0
Thick septa: 1
E- like changes: 0
* Interstitium:
Infl.   1
Fibrosis .   0
Congestion   2
**TOTAL: . 5/24
—---
** NAP
Male: [mean alv count: 12]
*278 M:
*Alveolar count: 13
*Alveoli.
Cell debris: .   1
Infl cells: 0
Fibrin:.   0
Thick septa: 1

E- like changes: 0
* Interstitium:
Infl. 2
Fibrosis . 0
Congestion. 2
**TOTAL: . 6 /24

*279 M:
*Alveolar count: 11
*Alveoli.
Cell debris: . 0
Infl cells: 0
Fibrin:. 0
Thick septa: 0
E- like changes: 1
* Interstitium:
Infl. 2
Fibrosis . 0
Congestion. 2
**TOTAL: . 5/24

281 M:
*Alveolar count: 12
*Alveoli.
Cell debris: . 0
Infl cells: 0
Fibrin:. 0
Thick septa: 0
E- like changes: 1
* Interstitium:
Infl. 2
Fibrosis . 0
Congestion. 2
**TOTAL: . 5/24

Female: [mean alv count: 12.66]
*274 F:
*Alveolar count: 14
*Alveoli.
Cell debris: . 0
Infl cells: 0
Fibrin:. 0
Thick septa: 1
E- like changes: 0
* Interstitium:
Infl. 1

Fibrosis . 0
Congestion. 2
**TOTAL: . 4/24

*275 F:
*Alveolar count: 11
*Alveoli.
Cell debris: . 0
Infl cells: 0
Fibrin:. 0
Thick septa: 1
E- like changes: 0
* Interstitium:
Infl. 2
Fibrosis . 0
Congestion. 1
**TOTAL: . 4/24

*276 F:
*Alveolar count: 13
*Alveoli.
Cell debris: . 0
Infl cells: 0
Fibrin:. 0
Thick septa: 1
E- like changes: 1
* Interstitium:
Infl. 1
Fibrosis . 0
Congestion. 2
**TOTAL: . 5/24.
—---
